# Molecular Modeling the Proteins from the *exo-xis* Region of Lambda and Shigatoxigenic Bacteriophages

**DOI:** 10.3390/antibiotics10111282

**Published:** 2021-10-21

**Authors:** Logan W. Donaldson

**Affiliations:** Department of Biology, York University, Toronto, ON M3J 1P3, Canada; logand@yorku.ca

**Keywords:** protein structure, bioinformatics, gene regulation, virus-host relationships, dark-matter

## Abstract

Despite decades of intensive research on bacteriophage lambda, a relatively uncharacterized region remains between the *exo* and *xis* genes. Collectively, *exo-xis* region genes are expressed during the earliest stages of the lytic developmental cycle and are capable of affecting the molecular events associated with the lysogenic-lytic developmental decision. In Shiga toxin-producing *E. coli* (STEC) and enterohemorragic *E. coli* (EHEC) that are responsible for food- and water-borne outbreaks throughout the world, there are distinct differences of *exo-xis* region genes from their counterparts in lambda phage. Together, these differences may help EHEC-specific phage and their bacterial hosts adapt to the complex environment within the human intestine. Only one *exo-xis* region protein, Ea8.5, has been solved to date. Here, I have used the AlphaFold and RoseTTAFold machine learning algorithms to predict the structures of six *exo-xis* region proteins from lambda and STEC/EHEC phages. Together, the models suggest possible roles for *exo-xis* region proteins in transcription and the regulation of RNA polymerase.

## 1. Introduction

Over the past forty years, enterohemorrhagic *E. coli* (EHEC) O157:H7 [1] and newer serotypes such as O104:H4 [2] have been responsible for numerous outbreaks of food poisoning throughout the world [3]. While EHEC infections outcomes are generally limited to gastrointestinal discomfort, some infections progress to hemolytic uremic syndrome, a blood-borne kidney disease with a 5% mortality rate. EHEC strains are lysogens of λ family bacteriophages that carry a gene, either *Stx1* or *Stx2*, that encodes Shiga toxin [4,5]. When the bacterial host experiences stress, the dormant bacteriophage genome embedded within the host genome is excised and transcribed leading to a transition from the lysogenic to the lytic stage of development. The release of new viral progeny is accompanied by the release of Shiga toxin into the surrounding human intestinal tissues. Upon entering endothelial cells, the *N*-glycosidase activity of Shiga toxin cleaves 60S ribosomal RNA leading to a suspension of protein synthesis and eventual cell death. Most of the progress at controlling EHEC infection has been achieved through prevention by rigorously monitoring food and water sources that come in contact with livestock, a natural reservoir [6]. Antibiotic treatment of EHEC infections, unfortunately, is counterproductive because drugs that produce a stress response may stimulate the lysogenic-lytic transition and the consequent production of Shiga toxin [7,8,9].

Comparative genomics studies have helped reveal the wide diversity that is possible among Shiga-toxin producing bacteriophages (Stx^+^ phages). This diversity is likely accompanied by a myriad of ways that Stx^+^ phages may affect the virulence of their bacterial host. For example, the Stx^+^ phage Φ24_B_ is capable of increasing antibiotic resistance by reconfiguring the host metabolism [10]. In a mouse model, the introduction of Stx^+^ phage 933W into a non-pathogenic *E. coli* strain is sufficient to produce highly lethal infection [11]. A greater number of DNA replication origins may help the Stx^+^ phage P27 fine tune the lysogenic-lytic developmental decision in response to its microenvironment [12]. In contrast to the bacterium-phage relationship, the human-phage relationship is less characterized but nevertheless, could be an important factor to consider during infection [13]. For example, during *S. aureus* infections, a phage lysin CBD is able to weaken its hosts on two fronts by simultaneously repressing the expression of bacterial virulence genes and increasing the activity of human macrophages [14]. Aside from Shiga toxin, no other proteins have been identified in Stx^+^ phages that modify the human host. Nevertheless, continuing research into human-phage relationships may help drive new therapeutic strategies [15].

A set of relatively uncharacterized open reading frames (ORFs) between the exonuclease gene (*exo*) and the excisionase gene (*xis*) in lambdoid bacteriophages has been shown to affect the lysogenic-lytic developmental decision. These so-called *exo-xis* region genes tend to produce a greater effect in Stx^+^ phages than λ, the most studied archetype and logical reference for comparison. Since Stx^+^ phages are also known to be more sensitive to prophage induction, the *exo-xis* region represents a new focal point for understanding how Stx^+^ phages adapt to a rapidly changing microenvironment in their hosts and in the human gut [16].

In bacteria expressing additional copies of the *exo-xis* region, the overall survival rate is lower [17]. Additional copies of the *exo-xis* region also increase viral DNA content, corroborating a link to DNA replication that was noted in the first study of the λ *exo-xis* region [18]. Expression of RecA-dependent SOS response genes are reduced during infection with phages bearing a deletion of the *exo-xis* region [19]. Since oxidative stress, antibiotic treatment and UV-induced DNA damage all lead to an SOS response [20], the *exo-xis* region may help the bacteriophage modulate a broad range of stress-associated inputs from its host [21]. The suppressive action of gene expression by the *exo-xis* region against the bacterial host also affects the transcription of lytic genes [22]. 

In this article, I begin by describing the genomic organization of the *exo-xis* region and then delve further into the structural and functional features of each gene by reviewing the current literature and presenting new molecular models of each gene product if an experimentally determined structure is not known. Further explorations of the *exo-xis* region have the potential to reveal new insights into the lysogenic-lytic developmental decision and new therapeutic routes to combat infection.

## 2. Conserved Genes of the *exo-xis* Region

**Figure 1:**
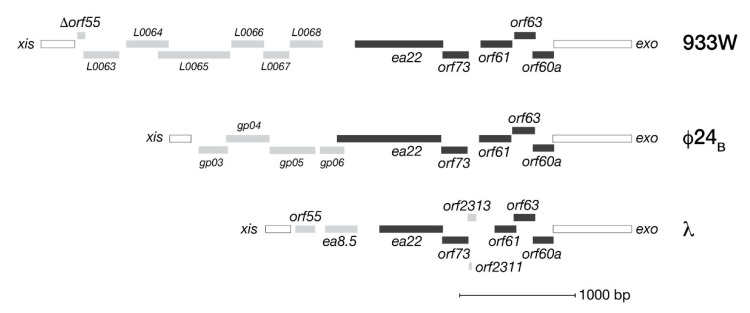
The *exo-xis* regions of λ and two Shiga toxin producing phages (933W, Φ24_B_) associated with clinically relevant *E. coli* infections. Solid bars indicate a typically conserved core set of genes. Other genes are indicated by shaded bars.

By virtue of their location in the phage genome, *exo-xis* genes are among the earliest transcribed during infection from the *pL* promoter. Figure 1 presents an overview of the open reading frames (ORFs) comprising the *exo-xis* regions of bacteriophage λ and two model Stx^+^ phages, Φ24_B_ and 933W. In each phage genome, five genes consisting of *orf60a*, *orf63*, *orf61, orf73* and *ea22* form a conserved block followed by a variable set of downstream genes both in terms of number and kind. Originally predicted by bioinformatics, these ORFs have since been confirmed by ribosome profiling methods of bacteriophage λ as *bona fide* expressed genes [23]. In λ, ribosomal profiling has also identified two additional genes (*orf2311* and *orf2313*) in the same conserved region that encode peptides of 7 and 31 amino acids, respectively.

Most of the effects of the *exo-xis* region genes to date have been described by the Węgrzyn laboratory (Univ. Gdansk). Throughout their studies, they have used four functional assays to assign the behavior of a given *exo-xis* gene as either pro-lysogenic or pro-lytic [24,25,26,27]. Two of the assays examine the time it takes for the lytic cycle to begin either from the onset of infection or the induction of lysogens by stressors such as hydrogen peroxide, antibiotics, and ultraviolet light. The remaining two assays quantify the relative number of survivors that escape infection or continue as lysogens. Collectively the assays suggest *orf73* and *ea22* as pro-lysogenic genes while *orf60a*, *orf63* and *orf61* tend to be pro-lytic. Since all *exo-xis* genes are actively transcribed during early viral development, the interplay among viral proteins and host proteins could contribute considerable complexity to the lysogenic-lytic decision. To resolve this complexity, a systems biology approach incorporating contemporary genomics and proteomics approaches may offer a way forward.

## 3. The Interaction Landscape of *exo-xis* Region Proteins

### 3.1. Oligomerization and Phase Separation

The majority of *exo-xis* region proteins are less than one hundred amino acids in length, suggesting they are organized into one or two domains. Therefore, to achieve their function, the *exo-xis* region proteins may need to oligomerize or combine with a protein partner. One yet to be explored aspect of the lysogenic-lytic developmental decision is the role of liquid-liquid phase separation (LLPS). While LLPS is more established in eukaryotes as a means for proteins and protein complexes to coalesce into a functional unit resembling a membrane-less organelle, there are now several examples of this phenomenon in bacteria [28]. Two interesting examples of bacterial LLPS are the condensation of the Dps DNA-binding protein to protect the nucleoid from damage [29] and the condensation of single-stranded DNA-binding protein (SSB) to sequester proteins involved in DNA replication, DNA repair and recombination [30]. While LLPS has not been demonstrated for *exo-xis* proteins, it could provide a means for phage proteins to sequester host proteins in a rapid, concentration-dependent manner.

### 3.2. Yeast Two-Hybrid Studies

**Figure 2:**
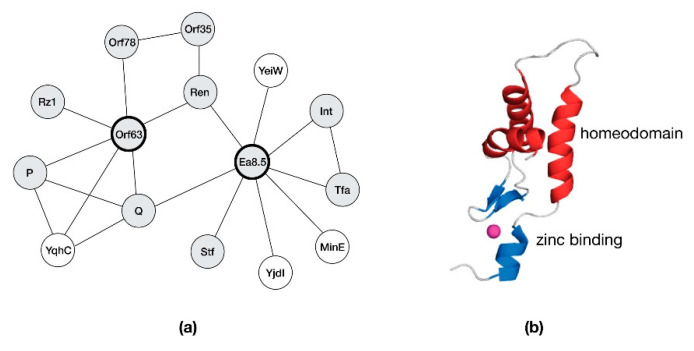
Potential interactions of *exo-xis* proteins identified from yeast two-hybrid studies. (**a**) An interaction map of host proteins (open circles) and phage proteins (shaded circles). (**b**) The NMR solution structure of Ea8.5 (PDB: 2M7A) reveals an unusual hybrid homeodomain-like/zinc-finger fold.

Host metabolic and stress response pathways are the most plausible targets of *exo-xis* proteins. Within those pathways, *exo-xis* proteins may exert wide ranging effects through interactions with RNA polymerase, regulatory sequences and response regulators. To date, potential protein partners for two λ *exo-xis* proteins have been identified as from a yeast two-hybrid (Y2H) study that screened λ phage protein baits with a library of either phage or bacterial protein preys [31,32]. In Figure 2, an interaction map is presented for Orf63, a protein conserved among λ and Stx^+^ phages, and Ea8.5, a protein exclusive to λ. The map shows that both Orf63 and Ea8.5 are associated with the Ren and Q as partners. Ren is a transcriptional activator that allows prophages to be superinfected by a similar phage but not by unrelated phages [33,34]. Q is an anti-termination factor that permits middle and late phage genes to be expressed by preventing the formation of pausing and terminator hairpins in the emerging RNA at the exit-channel of RNA polymerase (RNAP) [35]. Stf and Tta are tail-associated structural proteins and may not be true partners of Ea8.5. One promising interactor for Orf63 from the Y2H assay is YqhC, a transcription factor that stimulates expression of YqhD, an enzyme that amplifies the oxidative stress response. If Orf63 bound and inhibited YqhC, neutrophil mediated oxidative attack in the gut would be diminished thereby creating a more favorable environment for the phage to develop [24]. Turning to Ea8.5, the identification of MinE as a potential protein partner may suggest a role in altering the cell division process. Since MinE helps maintain the Z-ring assembly in the middle of the cell [36,37], Ea8.5 in turn, may sequester it or bias MinE towards one conformation. In support of this role, Ea8.5 is known from early functional studies for its ability to help synchronize cell division [18]; however, its structure suggests that it may serve as a transcription factor instead [38]. Further biochemical and biophysical studies with purified proteins and protein fragments are necessary to reveal the precise function role of Ea8.5 and other *exo-xis* proteins.

Aside from Orf63 and Ea8.5, no other *exo-xis* protein partners were identified by the Y2H studies. Since the Y2H method only identifies binary protein-protein interactions, it is possible that some *exo-xis* proteins participate in large protein assemblies. Alternatively, some *exo-xis* proteins may have a nucleic acid as a ligand instead of a protein. With these possibilities in mind, it would be worthwhile to extend the Y2H studies by using contemporary proteomic approaches that combine direct capture with mass spectrometry-based detection.

## 4. Structural Features of the Conserved *exo-xis* Region Proteins

### 4.1. Machine Learning Methods for Protein Structure Prediction

Bacteriophage genomes are known to contain a lot of ‘dark matter’—genes with no predicted structure or function [39,40]. Due to the vast evolutionary distance between phages and other life, homologous sequences can be difficult to identify. Assuming that a common function might be obscured by an uncommon sequence, it is helpful to look past sequence and towards higher orders of structures. Machine-learning methods such as AlphaFold [41] and RoseTTAFold [42] have enabled structure prediction of the entire human proteome at new levels of accuracy and detail [43]. These methods, however, can be challenging for bacteriophage proteins due to the fact that they use multiple sequence alignments to guide the process. The robustness of these methods to alterations in multiple alignment has only recently been explored [44]. Other methods that do not rely upon a multiple sequence alignment have been reported as well, but have not yet matured into a publicly accessible service [45]. All machine learning methods tend to have more demanding computational requirements (memory, database storage and specialized GPUs).

The accuracy of an AlphaFold or RoseTTAFold prediction is expressed as a pLDDT value (predicted Local Distance Difference Test) that is assigned on a per residue basis and ranges in value from 0 to 100 [46]. Residues in the model with pLDDT values between 70–100 are considered to have enough confidence to make statements about the backbone. Residues with pLDDT values between 50–70 should be considered more carefully. Low confidence should be assigned to residues with a pLDDT < 50 as this range also includes predicted disordered regions.

**Figure 3:**
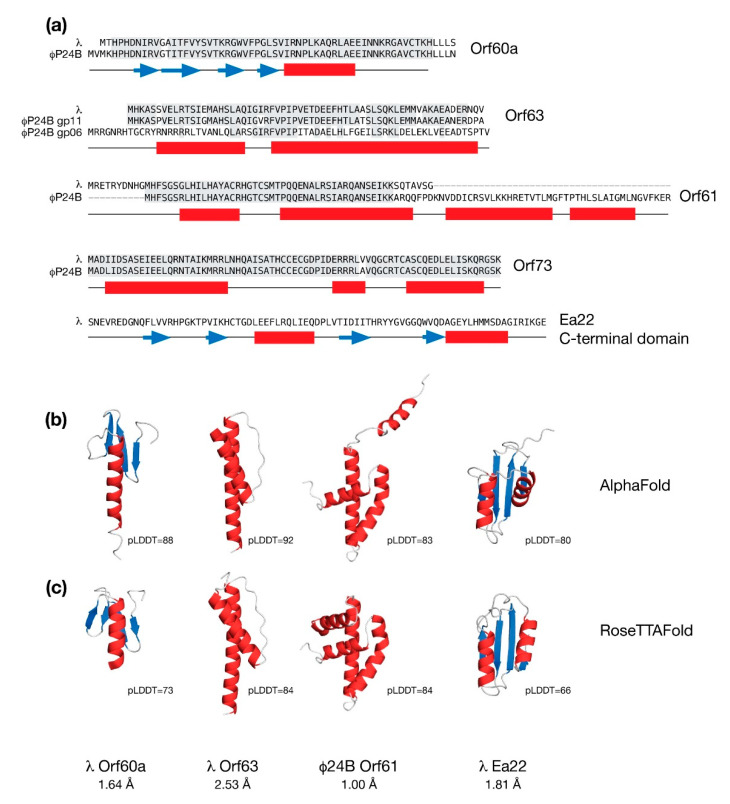
Molecular modeling the conserved *exo-xis* region proteins in their monomeric forms from bacteriophages λ and Φ24_B_. (**a**) Sequence alignments are shown for Orf60a, Orf63, Orf61, and Orf73. Identical amino acids shaded grey. There are two paralogs of Orf63 in Φ24_B_. Only the C-terminal domain of Ea22 is shown. The predicted secondary structure is presented below the sequences with helices in red and strands in blue. (**b**) Molecular models of selected *exo-xis* proteins by AlphaFold. (**c**) Molecular models of selected *exo-xis* proteins by RoseTTAFold. Structural similarity (as backbone RMSD) and accuracies (as pLDDT values) between the AlphaFold and RoseTTAFold models are indicated. Due to the structural dissimilarity, residues 1-23 are not included in the statistics of the Φ24_B_ Orf61 model.

In Figure 3, predicted structures of the common exo-xis proteins are presented along with sequence alignment between each protein from λ and the Stx^+^ phage Φ24_B_. With the exception of Ea22, the remaining proteins Orf60a, Orf61, Orf63 and Orf73 are short and likely form only one discrete domain.

### 4.2. The Orf60a Gene of λ and Stx^+^ Phages

After infection by phage bearing a deletion in *orf60a*, there are more bacterial survivors and more lysogens among them suggesting that *orf60a* serves a pro-lytic role during development. In support of this role, the induction of lysogens after activation of the SOS response by antibiotics or oxidative stress takes longer to proceed in *orf60a* mutants. All of these effects are more pronounced for Φ24_B_ *Δorf60a* mutants than λ *Δorf60a* mutants.

The AlphaFold and RoseTTAFold predictions for Orf60a superimpose well with a Cα backbone RMSD of 1.84 Å. Each model is described by three or four anti-parallel β-strands serving as a platform for one α-helix. Compared to the AlphaFold model, the helix in the RoseTTAFold model is shorter and does not extend past the β-sheet. Since a helix with no contact is likely to be unstable in the absence of a ligand, the RoseTTAFold model was chosen for further analysis.

When the predicted structure of Orf60a was compared to the protein structure database, it compared favorably to a family of small DNA binding domains (DBDs) that use a β-sheet to interact with the major groove [47,48,49,50]. This family included the GCC box DNA binding domain from *Arabidopsis* AtERF (PDB:1GCC, RMSD = 1.97 A; 8% identity), the bacterial cell division reactivation factor CedA (PDB:2BN8, RMSD = 2.16 A, 23% identity), the λ phage amino-terminal integrase DBD (PDB:1KJK, RMSD = 1.67 A, 11% identity), and the Tn916 transposon DNA binding domain (PDB:1B69, RMSD = 1.76 A, 3% identity).

**Figure 4:**
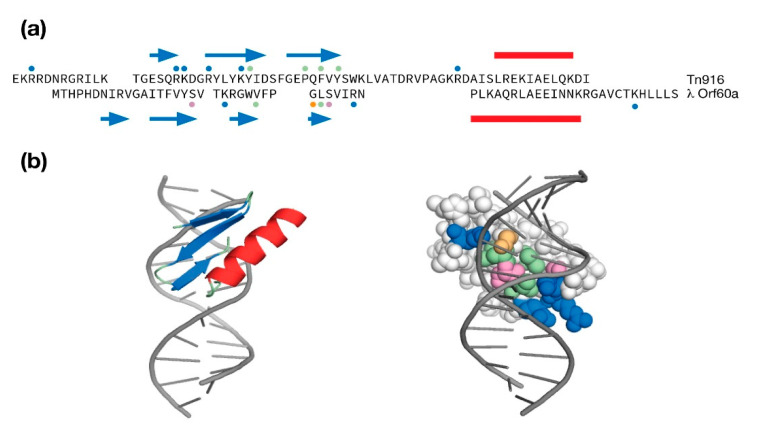
Modeling λ Orf60a as a potential DNA binding domain. (**a**) Structure based sequence alignment of λ Orf60a and the Tn916 DNA binding domain. An α-helix is indicated by a red bar, a β-strand is indicated as a blue arrow. Dots above and below the sequence indicate amino acids that make DNA contacts in the Tn916 NMR solution structure (PDB:1B69) or are predicted to make DNA contacts in the λ Orf60a model (pink = polar, green = aliphatic/aromatic, blue = basic). (**b**) The RoseTTAfold λ Orf60a model was docked onto DNA and refined to remove steric clashes. The space-filled protein is colored according to the legend below the λ Orf60a sequence.

Even though there was practically no sequence identity between the Tn916 structure and the λ Orf60a model, it was selected for a comparison because Tn916 was solved in its DNA-bound state. This comparison is presented as a sequence and secondary structure alignment in Figure 4a and as a refined λ Orf60/DNA model in Figure 4b. The λ Orf60a putative DNA binding domain uses all four β-strands to make DNA contacts with a mixture of basic amino acids at the periphery and polar/aliphatic amino acids within the major groove. While the complement of Tn916 contacts to DNA are different in their location within the sequence, they loosely follow the same pattern of basic amino acids at the periphery and aliphatic/aromatic amino acids within the groove.

While the true structure, function and mechanism of Orf60a is unknown, its potential role in gene expression aligns with its ability to serve the lytic program, especially in Stx^+^ phages.

### 4.3. The Orf63 Genes of λ and Stx^+^ Phages

As shown in Figure 3, φ24_B_ Orf63 is nearly identical in sequence to λ Orf63. However there is a second, less similar Orf63-like protein in f24B encoded by gene *gp06* that may have arisen due to a gene duplication event. Functional studies of phages bearing *orf63* deletions lead to role for this protein in the lytic developmental pathway. From Y2H studies (Figure 2), Orf63 may bind as many as six viral and host proteins which is surprising since like its namesake, Orf63 is only 63 amino acids in length.

AlphaFold and RoseTTAFold both predict that λ Orf63 is composed of two α-helices which is consistent with data obtained from a solution NMR study [24]. The experimental data also agree with the general location of the α-helices predicted by AlphaFold and RoseTTAFold; however, the experimental data demonstrate that the boundaries are much shorter. A size-exclusion chromatography coupled multi-angle laser scattering (SEC-MALS) analysis of purified λ Orf63 protein demonstrated that Orf63 is exclusively dimeric (unpublished observations).

While the accuracy scores are high for the two models of the Orf63 monomer, packing is very poor and there is less consensus on how the two α-helices cross (2.53 Å RMSD). Since AlphaFold supports the calculation of homo- and hetero-oligomers, another calculation was performed with homodimer selection. The highest scoring model (pLDDT = 72) was poorly packed and had the two helices twisted around each other by their intervening loops. As a result, this model was discarded. The sequence was then truncated to the folded boundaries that were identified by experimental data and AlphaFold was run again with a homodimer selection. Of the five structures calculated, each presented a different fold again with poor packing and poor accuracy (pLDDT = 24–29). From this study, it is clear that AlphaFold may be very sensitive to the length of the input protein, especially when the input sequence is short and the fold is oligomeric.

As a symmetrical, dimeric protein with four helices in total, Orf63 presents a very compact platform for potential protein partnerships. Further work is required to determine a high-resolution structure and identify protein partners.

### 4.4. The Orf61 Gene of λ and Stx^+^ Phages

The λ Orf61 protein appears to be truncated when compared to analogous proteins from the Stx^+^ phages Φ24_B_ and 933W. Both AlphaFold and RoseTTAFold analyses of λ Orf61 produced an extended structure with no discernible hydrophobic core suggesting a loss of structure and function. In contrast, the Stx^+^ phage Orf61 proteins were predicted to have a three-helix fold that strongly resembles a homeodomain. Thus, like Orf60a, Orf61 may serve to modify gene expression to promote the lytic developmental cycle [26]. As shown in Figure 3, the predicted models of Φ24_B_ Orf61 differ with respect to the positioning of an amino terminal helix, either contributing to the fold in the case of the RoseTTAFold prediction, or away from the fold in the case of the AlphaFold prediction. The remaining three α-helices superimpose very well with a backbone RMSD of 1.00 Å.

**Figure 5:**
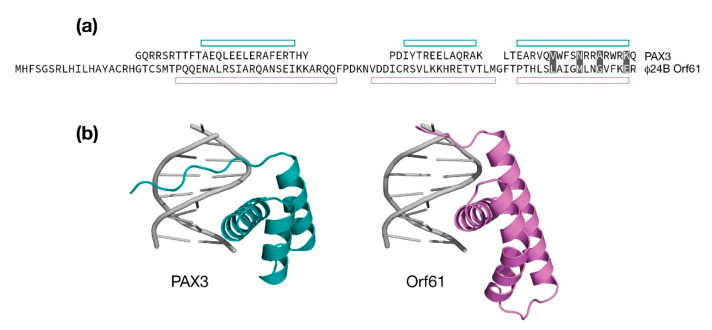
Modeling Φ24_B_ Orf61 as a potential DNA binding domain. (**a**) Structure based sequence alignment of Φ24_B_ Orf61 and the PAX homeodomain (PDB:3CMY). Open bars indicate the observed or predicted positions of three α-helices. Amino acids that make DNA major groove contacts are shaded. (**b**) Cartoon representation of the PAX3/DNA crystal structure (in teal). An amino-terminal extended segment reaches into the major groove. Cartoon representation of Φ24_B_ Orf61 model positioned analogously in the major groove.

The AlphaFold model (aa. 24–93) was searched against the PDB for similar structures. Several homeodomain folds were identified, one of which was human PAX3 in complex with DNA (PDB: 3CMY, RMSD: 1.83 Å, 4% identity) [51]. A comparison is presented in Figure 5. While the sequence identity is practically absent, the relative positioning of the three α-helices are consistent. The first two helices in the Φ24_B_ Orf61 model are longer and in the case of helix 2, slightly kinked at the point where it is extended relative to PAX3. A model of a Φ24_B_ Orf61 / DNA complex was made by replacing Orf61 with PAX3 and performing an refinement to remove steric clashes. A 3˚ bend in the PAX3 DNA structure was maintained in the Φ24_B_ Orf61 / DNA model. From the model of the complex, Orf61 is predicted to make four contacts from helix 3 to the DNA major groove. Since it is unknown if Φ24_B_ Orf61 is folded according to the model and binds DNA, it is not possible to speculate on the nature of the DNA sequence Φ24_B_ Orf61 may bind.

### 4.5. The Ea22 Genes of λ and Stx^+^ Phages

The ea22 gene provides some contrast to the three *exo-xis* genes that precede it. Not only is it the largest protein of the *exo-xis* region and multidomain in composition [52], but its function also favors lysogenic development over lytic development [27]. With this function in mind, Ea22 may serve as a new focal point for understanding how Stx^+^ phage lytic program can be delayed during EHEC infections to limit the release of Shiga toxin.

From sequence comparisons and experimental evidence derived from deletion studies, and biophysical analysis of purified proteins and protein fragments, λ Ea22 may be considered in terms of a variable *N*-terminal region, a central tetrameric coiled-coil region, and a dimeric C-terminal region. The C-terminal region demonstrates the hallmarks of a domain since it can be separated from the protein by limited proteolysis treatment while still retaining exceptional thermostability [52]. As shown earlier in Figure 3, a comparison of the AlphaFold and RoseTTAFold predictions for the λ Ea22 C-terminal domain as a monomer differ only in the positioning of the second helix with an overall backbone RMSD of 1.82 Å. The secondary structures, both in terms of length and position, were consistent with backbone chemical shift assignment data obtained from NMR solution studies the λ Ea22 C-terminal domain (unpublished observations). A search of the RoseTTAFold model of the λ Ea22 C-terminal domain against the PDB identified no homologs for comparison, either in a monomeric or dimeric form. The coiled-coil tetramerization domain was submitted to AlphaFold for oligomeric modeling; however, the resulting models neither identified a consensus fold nor were packed well.

**Figure 6:**
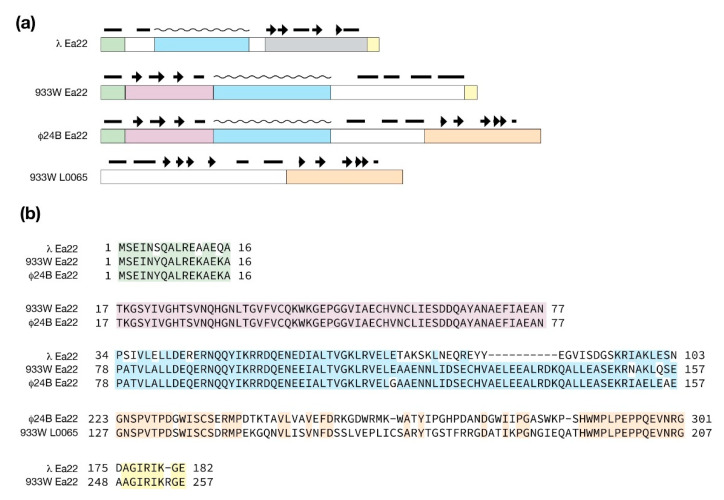
Ea22 proteins from λ and the Stx^+^ phages Φ24_B_ and 933W. (**a**) Blocks of similarity with predicted secondary structure above with helices (bars), strands (arrows) and coiled-coiled regions (wavy lines). (**b**) Sequence comparisons of the same blocks. The C-terminal sequence of λ Ea22 shaded grey cannot be aligned to the Ea22 sequences from Φ24_B_ and 933W.

The multidomain organization of Ea22 proteins is explored in Figure 6. Compared to λ Ea22, Φ24_B_ Ea22 contains two additional regions that flank a common coiled-coil region. What likely arose as a gene insertion event, the *ea22* gene of 933W retains the first three domains it has in common with Φ24_B_ Ea22 protein and leaves the remaining C-terminal domain encoded by the gene *L0065.*

### 4.6. The Orf73 Genes of λ and Stx^+^ Phages

Among the conserved *exo-xis* proteins, Orf73 can be structurally modeled with high confidence since it is very similar to TraR, a bacterial protein that acts as a global regulator of stress-related genes by interacting with second regulatory channel of σ^70^-dependent RNA polymerase (RNAP). TraR inhibits the expression of ribosomal RNAs and ribosomal proteins and stimulates genes associated with amino acid biosynthesis and amino acid transport without the requirement for a ppGpp cofactor that is produced during a stress response [53]. From high-resolution cryo-EM studies, TraR alters the kinetics of promoter-bound RNAP at multiple stages ultimately inhibiting or stimulating transcription from the basal level [54]. In λ and Stx^+^ phages, deletion of Orf73 resulted in lower survival rates upon infection and a lower efficiency of lysogenization both suggestive of a pro-lysogenic role during the phage developmental cycle [25]. Thus, as a TraR-like factor, Orf73 has the capacity to modify the expression of a wide repertoire of genes associated with the stress response through a direct interaction with RNAP [23].

**Figure 7:**
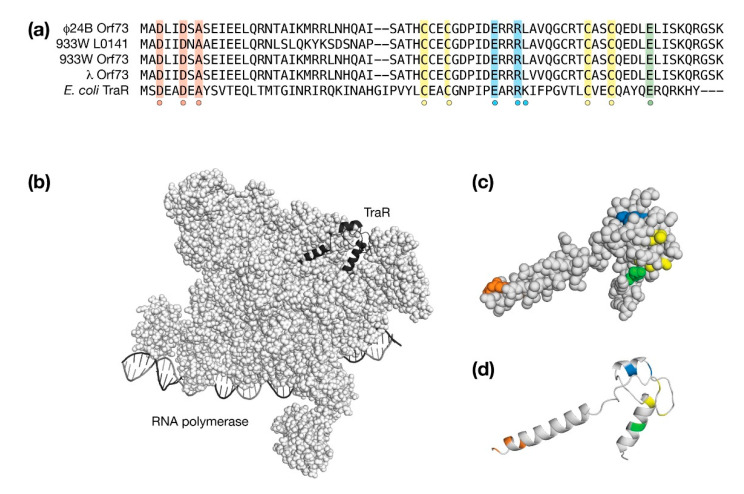
Molecular modeling the *exo-xis* protein, Orf73. (**a**) A sequence comparison of λ Orf73 and *E. coli* TraR. Similar and identical amino acids are shaded. A dot indicates conserved cysteines that coordinate one zinc ion. (**b**) Structure of the *E. coli* RNA polymerase (space filling representation), DNA (ribbon representation) and TraR (cartoon representation). (**c**,**d**) A ribbon and space filling representation of λ Orf73 following the same shading used in the sequence alignment.

Nearly all of TraR makes interactions with RNAP in an extended state [54]. The TraR structure may be considered in terms of a number of sequence hallmarks that are shared with λ Orf73 and the corresponding Orf73 proteins from the Stx^+^ phages φ24_B_ and 933W. Of note, within the phage 933W *exo-xis* region, there are two Orf73 sequences encoded by genes *orf73* and *L0141*, respectively. As shown in Figure 7a, the first sequence hallmark is an acidic DxxDxA motif at the beginning of the first helix. This acidic motif makes contacts deep within RNAP. In other proteins such as DksA [55], GreA [56], and Rnk [57], the amino terminal helix is substituted by a coiled-coil that presents the acidic amino acids at a tight turn. The second sequence hallmark is a C4 zinc binding motif that is conserved among all Orf73 proteins and TraR. The third sequence hallmark is an acidic amino acid in the carboxy terminal helix [53,58]. The fourth and final sequence hallmark ExxRK is required for interactions with the Si3 region of RNAP.

The AlphaFold and RoseTTAFold predicted models of λ Orf73 were very similar with a backbone RMSD of 0.92 Å. Despite this structural similarity, each had a different accuracy associated with them (AlphaFold pLDDT = 93, RoseTTAFold pLDDT = 57). An alignment of the best representative AlphaFold λ Orf73 model with TraR in its RNAP bound form aligned with a Cα RMSD of 4.02 Å due to the relative angles of the first and third α-helices. The AlphaFold and RoseTTAFold models may have revealed a requirement for a conformation change upon binding.

The large RMSD difference between the unbound Orf73 models and TraR made direct rigid-body docking ineffective. In lieu, a bound form of λ Orf73 was made directly from TraR, places coarsely into RNAP by substitution and then refined with the Rosetta 3.1 software package to minimize any steric clashes. The backbone RMSD change before and after refinement was 0.84 Å indicating only minor structural adjustments were required to dock λ Orf73 in RNAP. The λ Orf73/RNAP model is presented in Figure 7b. The λ Orf73 model in its bound form is presented in Figure 7b,c to highlight the sequence hallmarks it shares with TraR. From an examination of the λ Orf73/RNAP model, no potentially unsatisfied buried charges were observed. The TraR ExxRK motif deviates slightly for λ Orf73 as ExxRL; however, the leucine substitution in the λ Orf73/RNAP model appears to compensate for the aliphatic component of solvent-facing lysine sidechain in TraR.

## 5. Non-Conserved *exo-xis* Region Proteins of Interest

### 5.1. Bacteriophage λ Orf55 Is a Possible Modulator of RNA Polymerase

The final gene in the *exo-xis* region of λ bacteriophage is an uncharacterized ORF simply called *orf55*. There is no *orf55* homolog in the Stx^+^ phage φ24_B_; however, in phage 933W, there a portion of *orf55* at an analogous location near the *xis* gene. AlphaFold and RoseTTAFold both predicted a similar fold for Orf55 consisting of three β-strands and one α-helix (0.94 Å RMSD). A search against the PDB revealed three homologous structures: The first candidate was p56 (PDB: 4L5N), a uracil glycosidase inhibitor from *B. subtilis* phage φ29 [59]. While structurally similar (2.8 Å RMSD), it was only 5% identical at the sequence level. The second candidate (PDB: 4NJC; 2.1 Å RMSD) was the ε subunit of RNAP of gram-positive bacteria encoded by the gene, *rpoY* [60]. The ε subunit had no sequence identity with Orf55. The third and final candidate was Gp2 (PDB: 4LLG), an inhibitor of RNAP from phage T7 with an RMSD of 2.07 Å and an overall sequence similarity of 20% [61].

**Figure 8:**
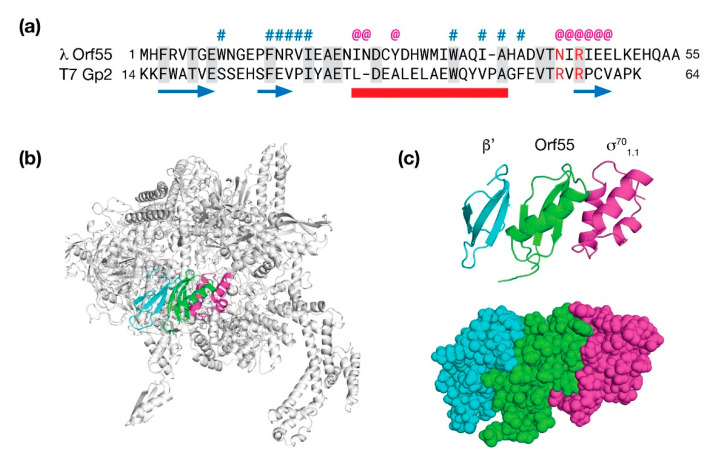
Molecular modeling λ Orf55. High confidence predictions by AlphaFold (pLDDT = 93) and RoseTTAFold (pLDDT = 93) both present Orf55 as a a small α/β protein. From a scan of the PDB for homologs, T7 Gp2 was identified as a candidate. (**a**) Sequence alignment of λ Orf55 and T7 Gp2. The predicted secondary structure is shown underneath the sequence (β-strands, blue arrows; α-helix, red bar). Sequence identity is denoted by shading. Above the sequence, a # indicates a predicted interaction with the RNAP β′ subunit and a @ indicates a predicted interaction with the 1.1 domain of the general sigma factor, σ^70^. (**b**) The Rosetta software suite was used to refine a molecular model of Orf55 (green) situated in RNAP. Numerous contacts are made between Orf55 in the model with the β′ subunit (blue) and σ^70^ (magenta). (**c**) Ribbon and space-filling diagram detail of Orf55 flanked by the RNAP β′ subunit and σ^70^.

The T7 phage Gp2 protein interacts with β′ subunit of RNAP and translocates a domain (1.1) of σ^70^ to block the downstream DNA duplex channel and effectively shut down host transcription in favor of transcription by T7 polymerase. To examine how Orf55 may bind RNAP in an analogous way to T7 phage Gp2 protein, a molecular model was made by substituting Orf55 for Gp2 in the Gp2/RNAP complex and then using the Rosetta software suite to make minor backbone moves to minimize steric clashes and to maximize favorable contacts. The results of this modeling study are presented in Figure 8.

In the Orf55/RNAP model, Orf55 is positioned between β′ and σ^70^_1.1_ and makes extensive contacts with both proteins. It is known that substitution of one of two arginines to alanine near the third β-strand of Gp2 are deleterious both in terms of RNAP inhibition (~20% of wild type) and affinity (~12% of wild type). The analogous amino acids in the Orf55 model are N43 and R45; thus, one amino is substituted and one is identical. Of the two positions, N43 is partially surface exposed and consequently, not necessarily incompatible with the complex. The second position, R45, is positioned deeper at the interface with the β′ subunit. Favorable ionic contacts in the model are observed with E47 of Orf55 and E1158 of the β′ subunit. At the other interface, a cluster of favorable hydrophobic contacts in the model are made between W9 of Orf55 and L9, I44, and I48 of σ^70^.

While this model is speculative, the presence of Orf55 during early stages of infection may serve an antagonistic role against σ^70^-mediated gene expression, assuming its affinity for RNAP is moderate. Expression of genes related to the stress response would remain unaffected. Remodeling the dynamics of gene expression, even transiently, could have far reaching effects in the bacterial host and the efficiency at which λ proceeds through lytic developmental pathway.

### 5.2. Bacteriophage φ24_B_ Gp05 Is a Possible Transcription Factor

In bacteriophage λ, two unique genes, *ea8.5* and *orf55*, are situated between *ea22* and *xis*. Both proteins have been presented in this report either as a solved structure (Ea8.5) or a high confidence molecular model (Orf55). Compared to λ, the Stx^+^ phages φ24_B_ and 933W harbor more genes in the *exo-xis* region along with instances of full or partial gene duplication. Here, a molecular model of the φ24_B_ *gp05* (*vb_24B_3c*) gene product is presented as an example of an *exo-xis* protein with a novel combination of features. It is currently unknown if *gp05* is expressed and encodes a functional protein.

**Figure 9:**
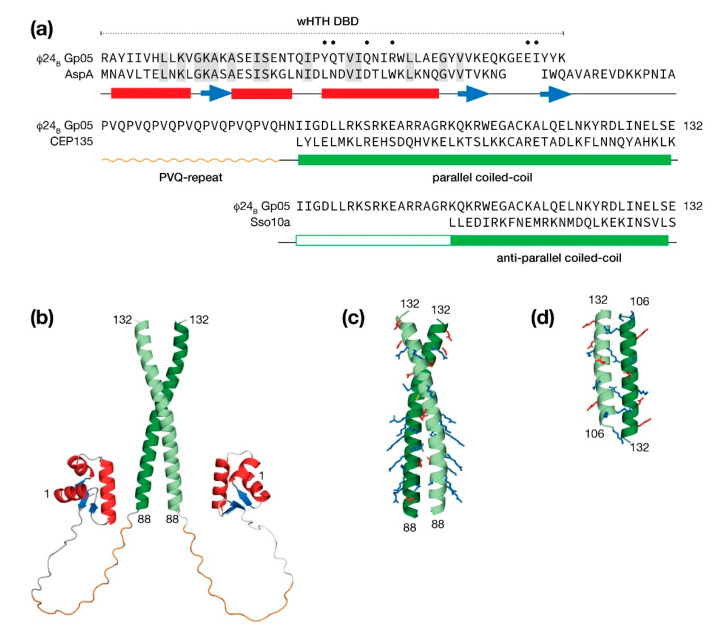
Molecular modeling the multidomain protein Gp05 from the Stx^+^ phage Φ24_B_. (**a**) The protein may be considered in terms of a winged helix-turn-helix (wHTH) DNA binding domain (DBD), a proline-valine-glutamine (PVQ) repeat region and a coiled-coil region. Predicted helices (bars) and strands (arrows) are shown below the sequence. An alignment with a similar wHTH domain from the AspA centromere binding protein (PDB: 5K5R) is shown for comparison. Sequence identity is denoted by shading, a dot indicates amino acids that are predicted to make DNA base contacts. Following the PVQ-repeat, the coiled-coil domain was modeled either as parallel coiled-coil spanning the entire predicted region against the human centriolar protein CEP135 (PDB:5FCN) or as an anti-parallel coiled-coil partially spanning the predicted region against the *Solfolobus* chromosomal maintenance protein Sso10a (PDB: 1R7J). (**b**) A representative full model of dimeric Gp05 determined by AlphaFold with a coiled-coil region in a parallel configuration. (**c**) A molecular model of the Gp05 coiled-coil domain in a parallel configuration determined using the Rosetta software suite. Charged amino acids are shown in sticks representation. (**d**) A molecular model of a partial Gp05 coiled-coil domain in an anti-parallel configuration determined using the Rosetta software suite.

The predicted structures by AlphaFold and RoseTTAFold both describe a protein that is composed of a winged helix-turn-helix (wHTH) DNA binding domain (DBD), followed by a PVQ-repeat leading into a carboxy-terminal coiled-coil domain (Figure 9a). Since the AlphaFold method is capable of modeling oligomers [62], the Gp05 was submitted for prediction as a homodimer.

From one representative AlphaFold model presented in Figure 9b, an extensive linker region in Gp05 protein would permit its wHTH DBDs to bind sequences spaced tens of bp apart. A search of the PDB for similar wHTH DBDs identified the hyperthermophilic archaeon *Sulfolobus* AspA chromosomal partitioning protein with 26% identity. The identical amino acids mapped closely to the hydrophobic core supporting the prediction of a wHTH DBD in the *N*-terminus of Gp05. The identical amino acids; however, did not include those in third helix that make base-specific contacts to DNA major groove suggesting that Gp05 binds a different sequence. In addition, the wing of Gp05 was shortened to the extent that adjacent minor groove specific contacts would likely not be possible. Thus, if DNA binding by Gp05 did occur, the binding site would be shorter than a typical wHTH domain.

A majority of the PVQ-repeat was modeled as a left-handed polyproline helix by AlphaFold, perhaps guided by numerous SH3 domain ligands that populate the PDB. A subsequent search of the PDB with BLAST revealed no proteins with a PVQ-repeat leaving the question of conformation unknown, notwithstanding a potential function.

The AlphaFold method selected a parallel configuration for the coiled-coil region. Closer examination of the dimeric interface indicated the the packing was poor and therefore the model had low confidence in this region. Consequently, a search of the PDB was made for dimeric parallel and anti-parallel coiled coil examples. A parallel coiled-coil candidate presented in Figure 9c was homology modeled from the human centriolar protein, CEP135 [63]. This quality of this model is supported by the facts that the CEP135 coiled-coil is the same length as the predicted coiled-coil Gp05 and the interface is well-packed with a combination of hydrophobic contacts and salt bridges. One striking feature of the Gp05 parallel coiled-coil model is a basic surface throughout the first half of the coiled-coil reminiscent of a leucine zipper DNA-binding motif [64]. An anti-parallel model was homology model from the *Sulfolobus solfataricus* Sso10a protein. Sso10a has a similar overall organization with Gp05 with an amino terminal wHTH DBD followed by a shorter linker leading into coiled-coil dimerization region. The Sso10a anti-parallel coiled-coil is well packed; but unlike CEP136, the Sso10a coiled-coil leaves a portion of the Gp05 unassigned (Figure 9d).

## 6. Discussion

Throughout the long history of bacteriophage λ research, the *exo-xis* region has remained largely uncharacterized mainly due to its dispensability for normal development in the laboratory. Recent work using ribosome display technology and other methods to measure gene expression have shown that *exo-xis* region genes are indeed expressed during the earliest stages of lytic development. In functional terms, *exo-xis* region proteins, alone or together, are able to affect bacterial cell cycle progression, DNA replication and the period in which lytic development occurs either through direct infection or by the induction of lysogens resulting from activation of SOS pathways. Using contemporary machine learning prediction methods, the molecular models presented in this report suggest that *exo-xis* proteins augment bacteriophage development by directly affecting gene expression through that action of transcription factors or indirectly affecting gene expression by modifying RNA polymerase. The *exo-xis* region of Stx^+^ phages is not only more extensive, but also highly mosaic suggesting that phage-host relationship in EHEC could be more complex.

While there is no doubt that AlphaFold and RoseTTAFold predictions are powerful new tools to identify new structures and relationships, these methods do have limitations. As shown in this study, there was no consensus between the methods of the multidomain protein Ea22 and the small oligomeric protein Orf63. While the vast sequence diversity of bacteriophages may have contributed to the low predictive success in cases, it may also offer a new way for these predictive methods to improve.

## 7. Materials and Methods

The genome reference sequences of bacteriophages λ, (NC_001416.1) Φ24_B_ (vB_EcoP_24B/NC_027984.1) and 933W (AF1225520.1) were used for this study. Molecular models of phage proteins were computed on the Google Colab cloud network using Jupyter notebooks [65] for the RoseTTAFold [66] and AlphaFold [67] methodologies. PDBeFold [68] was used to search the PDB for similar structures to the protein models described in this manuscript and perform pairwise Cα backbone alignments. PDBeFold was also used to substitute λ Orf55 with T7 Gp2 within RNAP (PDB: 4LLG) and to substitute λ Orf73 with TraR within RNAP (PDB:6PST). A large RMSD difference between the AlphaFold model of “free” λ Orf73 and “bound” TraR was observed. To mimic the bound form of λ Orf73, SWISS-MODEL [69] was used accompanied by a template sequence alignment.

Homology models of the λ Orf55/RNAP complex, Orf73/RNAP complex, and the dimeric coiled-coil of Φ24_B_ Gp05 against either CEP135 (PDB:5FCN) or Sso10a (PDB:1R7J) were made with the Relax module of the Rosetta 3.1 software suite [70] with options selected to suppress large domain motions and allow backbone motions that remain close to the starting structure. Secondary structure predictions were compared with those from AlphaFold/RoseTTAfold using the Quick2D module that is part of the HHPred software suite [71]. HHPred homology modeling was also used to suggest CEP135 as a parallel coiled-coil candidate for homology modeling.

## Data Availability

Data files of the molecular models presented in this study may be obtained from the YorkSpace Institutional Respository (http://hdl.handle.net/10315/38577, accessed on 1 September 2021).

## Abbreviations

DBD, DNA binding domain; EHEC, Enterohemorrhagic *E. Coli*; GPU, graphics processing unit; pLDDT, predicted Local Distance Difference Test; LLPS, liquid-liquid phase separation; ppGpp, guanosine pentaphosphate; PDB, Protein Data Bank; RNAP, RNA polymerase; RMSD, Root Mean Square Deviation; SEC-MALS, size-exclusion chromatography coupled multi-angle laser scattering; STEC, Shiga Toxin-producing E. coli, Stx^+^, shigatoxigenic bacteriophages; wHTH, winged helix-turn-helix.
